# Evaluating Dickkopf-1 as a biomarker: insights into periodontitis, rheumatoid arthritis, and their comorbidity—a systematic review and meta-analysis

**DOI:** 10.3389/fdmed.2025.1593218

**Published:** 2025-07-21

**Authors:** Bawatharani Maharavi, Jaideep Mahendra, Deepa Ponnaiyan, Vijayalakshmi Rajaram, Pragya Gyanchand, Roshan R. Rughwani, Kaustubh Suresh Thakare, Gayathri Kumar, Gauri Patil

**Affiliations:** ^1^ Department of Periodontics, Meenakshi Ammal Dental College and Hospital, Chennai, India; ^2^ Department of Periodontics, SRM Dental College, Ramapuram, Chennai, India; ^3^ Greatways Dental Clinic, Chennai, India; ^4^ Department of Periodontics, VYWS Dental College and Hospital, Amravati, India; ^5^ Department of Periodontology, SRM Institute of Science and Technology, Kattankulathur, India

**Keywords:** periodontitis, rheumatoid arthritis, pro-inflammatory biomarker, Dickkopf-1 (DKK-1), Wnt signaling pathway, bone remodeling

## Abstract

**Background:**

Dickkopf-1 is a glycoprotein that inhibits Wingless-related integration site signaling, impairing osteoblast and osteoclast functions, leading to bone loss and systemic inflammation linked to periodontitis and rheumatoid arthritis. *Porphyromonas gingivalis* exacerbates rheumatoid arthritis through citrullination and inflammation, highlighting their bidirectional relationship. To date no meta-analysis has examined the role of Dickkopf-1 in periodontitis, rheumatoid arthritis, and their comorbidity. Therefore, we conducted this meta-analysis to investigate the association and role of Dickkopf-1 in these comorbid conditions.

**Methods:**

The present study was conducted in accordance with the guidelines of Transparent Reporting of Systematic Reviews and Meta-Analyses PRISMA statement (registered at PROSPERO under the number CRD42025643227). A total of 15 studies (14 case–control and 1 cross-sectional) were selected out of 386 using databases like PubMed and Google Scholar (by BM, JM, and DP). A random-effects model evaluated Dickkopf-1 levels in serum/gingival crevicular fluid in periodontitis and rheumatoid arthritis via standardized mean difference (SMD) and 95% confidence intervals (CI). Heterogeneity and publication bias were assessed using statistical metrics, forest plots, funnel plots, Begg's test, and Egger's regression.

**Results:**

A total of 386 studies were retrieved and 15 were included in the meta-analysis, encompassing 4,438 participants (2,190 cases and 2,248 controls). The pooled SMD of 2.694 (*p* = 0.02; 95% CI: 1.170–6.203) indicated a significant association of Dickkopf-1 with periodontitis and/or rheumatoid arthritis compared to healthy controls. However, Egger's test revealed a t-value of 3.05 (*p* = 0.009), indicating significant publication bias.

**Conclusion:**

Elevated Dickkopf-1 levels in rheumatoid arthritis and periodontitis patients suggest its critical role in the pathogenesis of both conditions. Hence, Dickkopf-1 holds therapeutic potential for managing interconnected inflammatory and bone disorders and may serve as a biomarker for diagnosing these diseases.

**Systematic Review Registration:**

https://www.crd.york.ac.uk/PROSPERO/search, PROSPERO CRD42025643227.

## 1 Introduction

Periodontitis is an inflammatory disease of the tooth-supporting structures, leading to periodontal breakdown, alveolar bone destruction, root exposure, and tooth mobility (1). It is a multifactorial condition influenced by pathogenic bacteria, plaque, calculus, genetics, environmental factors, health conditions, pregnancy, lifestyle, and immune responses (2, 3) Biomarkers, chemical mediators, and signaling molecules drive inflammation causing tissue damage, pocket formation, and further destruction (4–6). Periodontal pathogens and pro-inflammatory mediators can enter systemic circulation (7), contributing to bacteremia, toxemia, and systemic conditions like cardiovascular disease (8), diabetes (9), adverse pregnancy outcomes (10), respiratory diseases (11), chronic kidney disease (12), and rheumatoid arthritis (13), potentially triggering or exacerbating these conditions (14). Rheumatoid arthritis is a chronic autoimmune disease that mainly leads to joint inflammation and pain. This may further be influenced by genetic and environmental factors (15, 16). Studies have identified periodontal bacteria, such as *Porphyromonas gingivalis*, *Prevotella intermedia*, *Prevotella melaninogenica*, *Tannerella forsythia*, and *Aggregatibacter actinomycetemcomitans* in the synovium of patients with rheumatoid arthritis (17, 18) These findings, along with elevated serum antibody levels against these bacteria, suggest that periodontal bacteria or their DNA may translocate from periodontal tissues to the synovium, exacerbating joint inflammation (19).

Dickkopf proteins are glycoproteins that are secreted from tissues and cells and antagonize the Wingless-related integration site (Wnt) signaling pathway, which plays a crucial role in bone biology by regulating osteoblastic and osteoclastic activities and bone formation (20). The inactivation of Wnt signaling disrupts bone homeostasis, leading to bone resorption (21). Comprising four key members—Dickkopf-1, 2, 3, and 4—these proteins feature an N-terminal soggy domain and two conserved cysteine-rich domains (CRDs) encoded by the Dickkopf gene (22, 23). Detected in various tissues, including bone, skin, gingiva, and serum, Dickkopf proteins are associated with systemic diseases, such as autoimmune disorders, neurodegenerative diseases, cardiovascular diseases, diabetes, periodontitis, rheumatoid arthritis, and other bone conditions (24).

Dickkopf-1, first reported by Glinka et al. in 1998, plays a significant role in the shared pathogenesis of periodontitis and rheumatoid arthritis, two chronic inflammatory diseases linked by systemic interactions (25). Overexpressed in the periodontium of periodontitis patients, Dickkopf-1 blocks the Wnt signaling pathway, promoting bone loss by disrupting osteoblastic and osteoclastic activity (26). Elevated serum levels of Dickkopf-1 in periodontitis patients with early rheumatoid arthritis further link it to dysfunction in bone metabolism and pathological bone resorption (27). Both diseases are characterized by elevated pro-inflammatory cytokines, such as TNF-α, IL-1β, and IL-6, driving inflammation and tissue destruction (28–30). Periodontal pathogens such as *P. gingivalis* are implicated in rheumatoid arthritis pathogenesis through citrullination and systemic inflammation, highlighting a bidirectional relationship (17, 18). Neutralizing Dickkopf-1 has shown potential in enhancing bone regeneration; clinical evidence suggests that managing periodontitis can reduce systemic inflammation and improve rheumatoid arthritis outcomes. Addressing their comorbidity is crucial for mitigating disease progression and enhancing patient care (31).

To date, no systematic review has explored the relationship between Dickkopf-1 in periodontitis, rheumatoid arthritis, or their comorbidity. Although elevated Dickkopf-1 levels have been reported in various inflammatory diseases, its role in periodontitis and rheumatoid arthritis remains unclear. Therefore, the objective of this systematic review and meta-analysis was to investigate the association and contribution of Dickkopf-1 in periodontitis, rheumatoid arthritis, and their comorbidity. The clinical question (PICO question) guiding this review was whether Dickkopf-1 levels differ between patients with periodontitis and/or rheumatoid arthritis (participants) and healthy controls (comparators), based on measurements of Dickkopf-1 in serum or gingival crevicular fluid (GCF; outcome) using enzyme-linked immunosorbent assay (ELISA) (intervention).

## 2 Methodology

This study was performed according to Preferred Reporting Items for Systematic Reviews and Meta-Analyses (PRISMA) guidelines (32). The protocol was registered *a priori* in PROSPERO (CRD42025643227) and aimed to analyze the Dickkopf-1 level in serum and GCF in relation to periodontal disease and rheumatoid arthritis. The target search strategy was prepared based on the research question, the articles to be analyzed were selected in accordance with the inclusion and exclusion criteria of the research, and the data were extracted and analyzed after quality evaluation.

### 2.1 Search strategy

**Figure 1:**
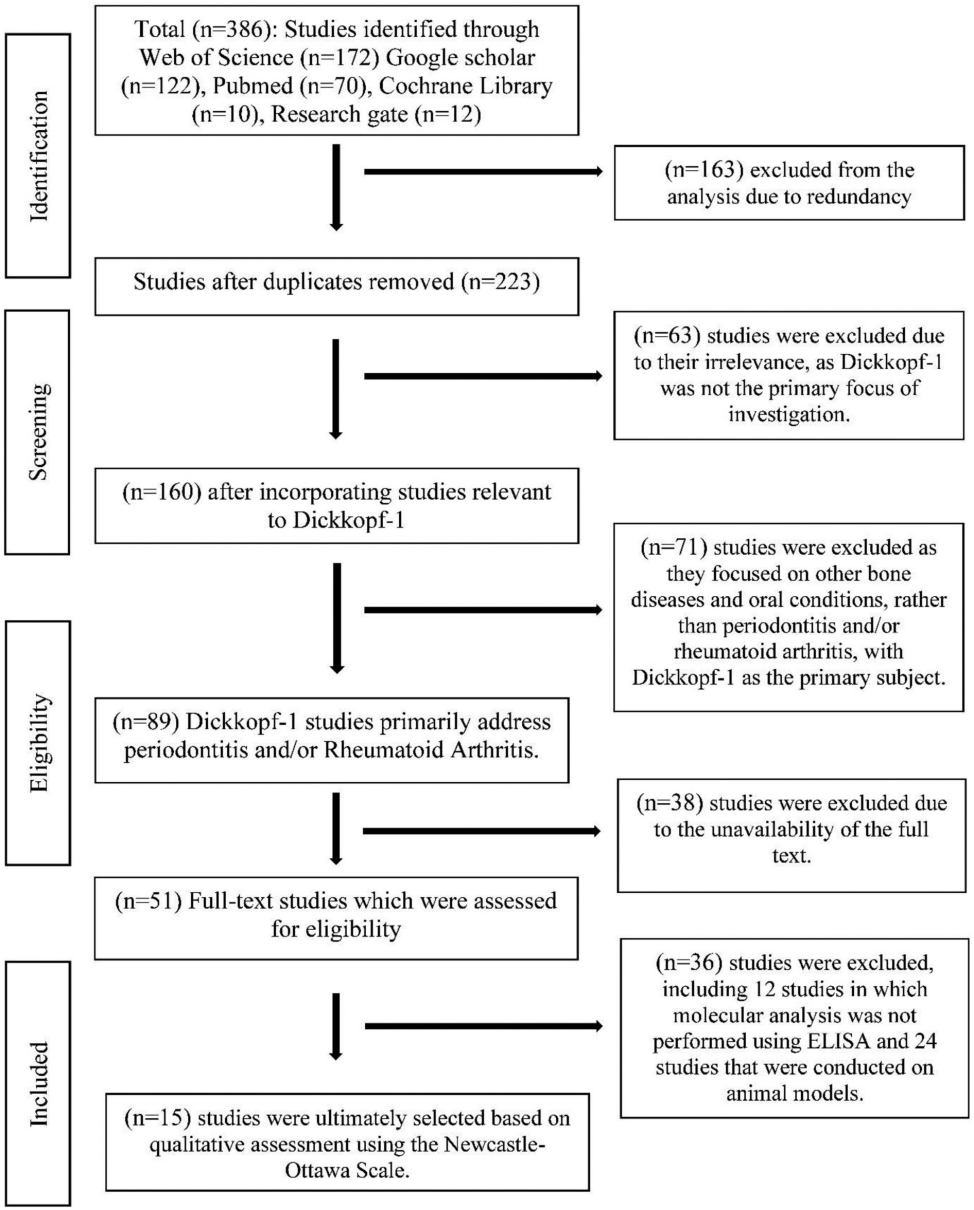
PRISMA flowchart of literature retrieval and study selection process.

The structured electronic search was conducted by three authors (BM, JM, and DP) independently across multiple databases between 1 June and 31 December 2024. Relevant articles published between 2009 and 2022 were identified and included in the analysis. In addition to structured searches in bibliographic databases (PubMed, Web of Science, and Cochrane Library), gray literature was screened using Google Scholar and ResearchGate. These platforms were employed to identify potentially relevant unpublished, preprint, or non-indexed studies. However, given their lack of standardized indexing and formal peer-review mechanisms, findings from these sources were interpreted with caution. MeSH terms were used and the search strategy was detailed in Figure 1. In addition, pertinent studies were manually searched from the references of reviews and enrolled studies. The following MeSH terms were used for searching: Rheumatoid Arthritis, Arthritis, Chronic Periodontitis, Periodontitis, Periodontal disease, Dickkopf-1, and Dkk-1. Combining these terms with logical operators such as AND or OR, search phrases like Rheumatoid Arthritis“[MeSH]” AND [“Chronic Periodontitis”[MeSH] OR “Periodontitis”[MeSH] OR “Periodontal Diseases”[MeSH]] AND “Dickkopf-1 Protein”[MeSH] OR “DKK-1”[MeSH]) were used to identify relevant studies for the meta-analysis (Supplementary File S1).

### 2.2 Eligibility criteria

The electronic search, literature search, and study selection involved an initial screening of studies published between June and December 2024, using specified search terms and assessing full-text availability. Corresponding authors were contacted when necessary. The authors independently screened titles and abstracts, excluding studies based on two criteria: (1) molecular analysis not performed using ELISA and (2) studies involving animal models. The remaining studies were reviewed for full-text inclusion with disagreements resolved by discussion. The final set of relevant studies met the following inclusion criteria: (1) case–control or cross-sectional design, (2) case groups of periodontitis and/or rheumatoid arthritis patients, (3) serum or GCF Dickkopf-1 levels measured in both case and control groups, (4) ELISA-based molecular analysis, and (5) exclusion of non-English articles, case reports, mechanistic studies, animal studies, non-ELISA molecular analysis, and review articles.

### 2.3 Evaluation of methodological quality, data extraction, and review questions

All 15 studies were initially screened based on their titles and abstracts by authors. The methodological quality of the studies and the sources of bias were assessed using the Newcastle–Ottawa Scale for case–control studies (33). Information from all databases was exported to an Excel spreadsheet (Microsoft Office 2013). The review question of this study, based on the PICOS framework, focused on the comparison of Dickkopf-1 levels in serum or GCF in systemically and periodontally healthy individuals [controls/comparator, C), with the population comprising patients with periodontitis, rheumatoid arthritis, or both conditions (comorbidity, P). The intervention/exposure included the presence of inflammatory conditions such as periodontitis, RA, or both, as these are linked to elevated Dickkopf-1 levels (I). The outcome assessed was the mean concentration of Dickkopf-1 in serum or GCF (O). The study design included observational studies (case–control or cross-sectional) that utilized ELISA as the detection method for Dickkopf-1 (S).

### 2.4 Data items

For subsequent analyses, the authors independently extracted the following data from the eligible studies: first author's name, year of publication, country, type of study, sample type (serum, GCF), molecular analysis method (ELISA), Dickkopf-1 levels, and the total number of participants, including controls and periodontitis and/or rheumatoid arthritis patients. Discrepancies in study selection, quality assessment, and data extraction were resolved through consensus discussions among the authors.

### 2.5 Statistical analysis

The analysis includes 15 studies, using the odds ratio (OR) as the effect size index. A random-effects model was employed, assuming that the selected studies represent a random sample from a broader pool of potential studies, with the results generalizable to this larger population (34). The relationship between serum or GCF Dickkopf-1 levels in patients with periodontitis and/or rheumatoid arthritis was assessed using the standardized mean difference (SMD) and 95% confidence interval (CI), incorporating the random-effects model to account for study heterogeneity (35). For all outcomes, the mean difference was used as the effect measure, with its corresponding confidence interval. A prediction interval was calculated to estimate the true effects. Statistical significance of the pooled SMD between patients and controls was evaluated using a *Z*-test. Heterogeneity across studies was assessed using the Q-statistic, *I*² statistic, Tau, Tau^2^, and forest plots (36). Publication bias was examined qualitatively and quantitatively using funnel plots, Begg's Mazumdar rank correlation test, and Egger's linear regression (37). All data analyses were conducted using Comprehensive Meta-Analysis version 4.

## 3 Results

### 3.1 Overview of studies included

#### 3.1.1 Number of studies

A total of 386 studies were retrieved from Web of Science (*n* = 172), Google Scholar (*n* = 122), PubMed (*n* = 70), Cochrane Library (*n* = 10), and ResearchGate (*n* = 12) between 1 June and 31 December 2024 (Figure 1). Among the 386 studies, 15 studies were finally included in the meta-analysis, based on the inclusion and exclusion criteria, representing data from 4,438 participants (2,190 cases and 2,248 controls).

#### 3.1.2 Study characteristics

The characteristics of the 15 included studies, consisting of 14 case–control studies and one cross-sectional study, are summarized in Table 1. These studies were conducted across Asia, Europe, and America and were published between 2009 and 2022. The included studies demonstrated moderate to high methodological quality based on the Newcastle–Ottawa Scale, with scores in the range of 4–7. Most studies adequately addressed participant selection and comparability, though a few showed limitations in outcome assessment. These quality differences were considered during interpretation, particularly regarding heterogeneity and the strength of evidence in studies with lower scores (Supplementary File S2). Among the studies, assessing the Dickkopf-1 levels in both serum and GCF, four compared Dickkopf-1 levels between periodontitis patients and healthy controls, one examined Dickkopf-1 levels in healthy controls and periodontitis associated with rheumatoid arthritis, and 10 focused on comparing Dickkopf-1 levels between rheumatoid arthritis patients and healthy controls. The total population data revealed that approximately 1,968 rheumatoid arthritis patients showed increased serum Dickkopf-1 levels compared to 1,821 healthy controls, and 45 rheumatoid arthritis patients showed decreased serum Dickkopf-1 levels compared to 50 healthy controls. Apart from this, 81 periodontitis patients had increased GCF Dickkopf-1 levels when compared to 61 healthy controls, and 33 periodontitis patients had decreased serum Dickkopf-1 levels when compared to 30 healthy controls. In addition, 63 patients with periodontitis along with rheumatoid arthritis showed increased serum Dickkopf-1 levels, compared to 286 healthy controls.

**Table 1:** Characteristics of included studies.

Sl. no	Author (year)	Country	Type of study	Type of sample	Total no of subjects		Dickkopf-1 levels		Newcastle–Ottawa Scale quality score
					Cases	Controls	Cases	Controls	
1	Cardona-Rincón et al. (38)	Colombia (SA)	Case–control	Serum	63	286	136.6 ± 58.03 ng/mL	9.10 ± 6.1 ng/mL	4
2	Antohi et al. (39)	Romania (E)	Case–control	Serum	33	30	1.209 ± 0.110 ng/mL	1.712 ± 0.100 ng/mL	7
3	Jin et al. (26)	China (A)	Case–control	GCF	30	10	8.86 ± 4.07 μg/L	5.51 ± 2.05 pg/μL	4
4	Wu et al. (40)	China (A)	Case–control	GCF	11	11	307.09 ± 45.63 μg/L	305.33 ± 147.40 μg/L	6
5	Jia et al. (41)	China (A)	Case–control	GCF	40	40	13.70 ± 3.62 μg/L	7.41 ± 2.02 μg/L	6
6	Nocturne et al. (42)	United States (NA)	Cross-sectional	Serum	708	974	30.03 ± 15.5 pmol/L	11.6 ± 4.2 pmol/L	6
7	Wang et al. (43)	China (A)	Case–control	Serum	100	40	5,952.6 ± 3019.5 pg/mL	3,198.9 ± 2283.6 pg/mL	7
8	Seror et al. (44)	France (E)	Case–control	Serum	694	453	28 ± 13.2 pmol/L	10.3 ± 9.4 pmol/L	6
9	Daoussis et al. (45)	Greece (E)	Case–control	Serum	45	50	1,845 ± 184.4 pg/mL	2,375 ± 123.8 pg/mL	6
10	Fassio et al. (46)	Italy (E)	Case–control	Serum	28	35	44.51 ± 17.81 pmol/L	27.29 ± 11.48 pmol/L	6
11	Świerkot et al. (47)	Poland (E)	Case–control	Serum	27	12	1,821.32 ± 1060.28 pg/mL	548.52 ± 36.35 pg/mL	6
12	Rossini et al. (48)	Italy (E)	Case–control	Serum	154	125	172 ± 68 pmol/L	96 ± 55 pmol/L	6
13	Choi et al. (49)	Republic of Korea (A)	Case–control	Serum	102	39	3,144.6 ± 1,718.7 pg/mL	1,369.9 ± 1,100.1 pg/mL	6
14	Long et al. (50)	China (A)	Case–control	Serum	100	50	3,760.88 ± 3,043 pg/mL	1,270.12 ± 716.42 pg/mL	6
15	Voorzanger-Rousselot et al. (51)	France (A)	Case–control	Serum	55	93	22,070 ± 18,073 pg/mL	3,724 ± 1,179 pg/mL	4

A, Asia; E, Europe; SA, South America; NA, North America; GCF, gingival crevicular fluid.

### 3.2 Overall effect size analysis

The pooled effect size was calculated as a standardized mean difference (SMD) of 2.694 [*p*-value = 0.02; 95% CI: 1.170–6.203] across the 15 included studies, indicating a significant association between periodontitis and/or rheumatoid arthritis in comparison to the control group. All 15 studies were analyzed to assess the strength of association between Dickkopf-1 levels in periodontitis and/or rheumatoid arthritis compared with controls. In total, 12 studies showed an OR >1 with a *p*-value <0.05, indicating statistical significance. However, the studies by Antohi et al. (39) and Daoussis et al. (45) had an OR <1. Wu et al. (40) had an OR of 1 and *p*-value >0.05, suggesting the result was not statistically significant (Table 2).

**Table 2:** Mean effect size analysis of included studies.

Model	Reference	Statistics for each study			
		OR	CI	Z-value	*p-*value
	Cardona-Rincón et al. (38)	3.926	2.369–6.505	5.307	0.000
	Antohi et al. (39)	0.000	0.000–0.001	−9.654	0.000
	Jin et al. (26)	5.183	1.347–19.939	2.394	0.017
	Wu et al. (40)	1.030	0.226–4.689	0.038	0.970
	Jia et al. (41)	49.013	18.071–132.938	7.645	0.000
	Nocturne et al. (42)	23.767	19.349–29.193	30.197	0.000
	Wang et al. (43)	5.837	2.909–11.713	4.965	0.000
	Seror et al. (44)	15.030	11.803–19.139	21.978	0.000
	Daoussis et al. (45)	0.002	0.001–0.006	−10.603	0.000
	Fassio et al. (46)	8.464	3.191–22.449	4.291	0.000
	Świerkot et al. (47)	13.420	3.440–52.360	3.739	0.000
	Rossini et al. (48)	9.071	5.695–14.447	9.285	0.000
	Choi et al. (49)	7.731	3.798–15.733	5.641	0.000
	Long et al. (50)	5.995	3.135–11.463	5.415	0.000
	Voorzanger-Rousselot et al. (51)	6.719	3.534–12.774	5.811	0.000
Random		2.694	1.170–6.203	2.329	0.020

OR, odds ratio; CI, confidence interval.

### 3.3 Heterogeneity

Based on the fixed random model analysis for heterogeneity among the 15 studies, it was observed that the Q-value = 473.9, *p* = 0.000 with a degree of freedom (d.f.) of 14, and *I*^2^ = 97%, indicating a substantial heterogeneity across the studies and suggesting that variability in study outcomes was higher than expected by chance alone. Further in the random model analysis, the Tau = 0.868 and Tau^2^ = 0.753 values indicated low variance between the studies. This suggests that the studies included in the meta-analysis were relatively heterogeneous among the included studies, indicating substantial variability in effect sizes across studies (Table 3).

**Table 3:** Meta-analysis model and heterogeneity statistics.

Meta-analysis model	Between-study SD (Tau)	Between-study variance (Tau^2^)	Cochran's Q (Q-value)	Degrees of freedom (df)	Significance (*p*-value)	Heterogeneity (*I*^2^, %)
Fixed random	0.868	0.753	473.908	14	0.000	97.046

#### 3.3.1 Forest plot

**Figure 2:**
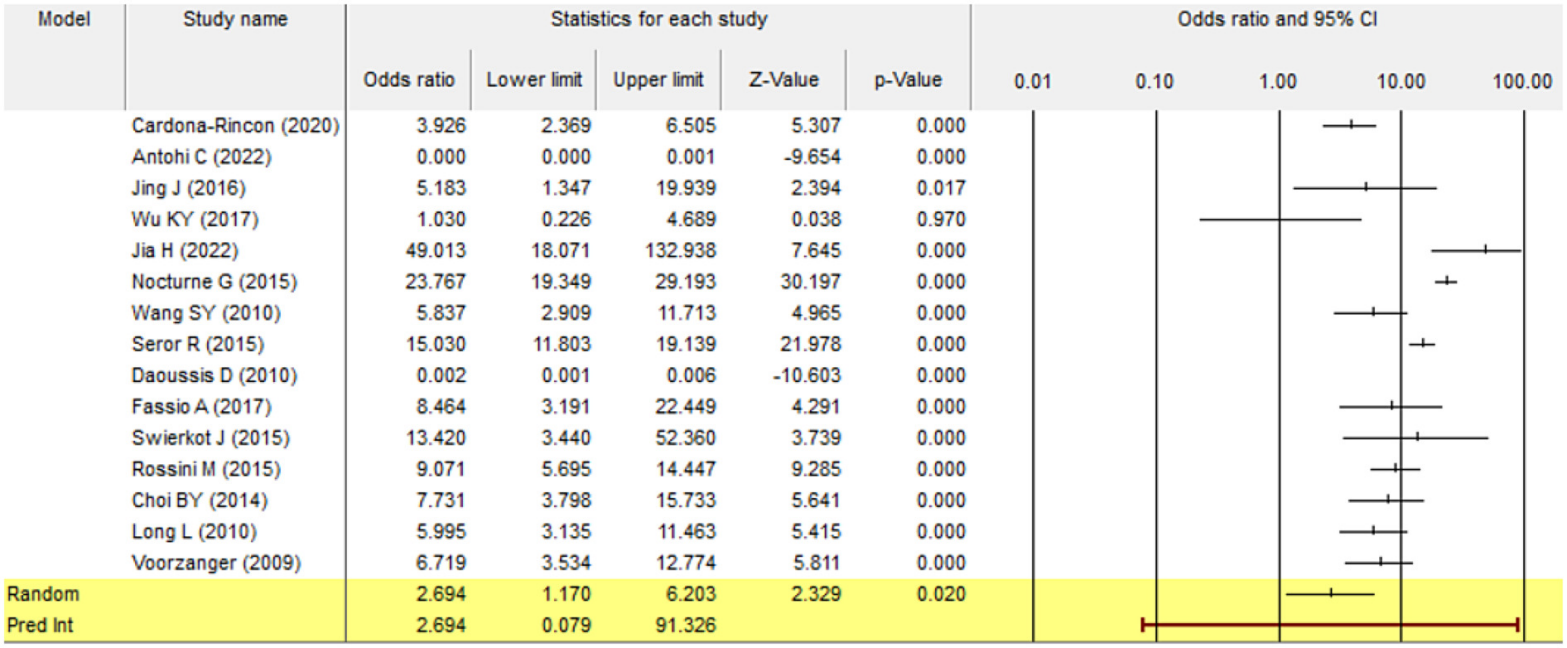
Flowchart of literature retrieval and study selection process.

A forest plot represents the individual studies by horizontal lines and their respective confidence intervals. The pooled effect size, represented by the diamond shape at the bottom, indicates a statistically significant positive effect with a 95% confidence interval (1.170–6.203) and *p*-value of 0.020. Most individual study results were consistent with the pooled estimate, suggesting low heterogeneity; however, certain studies showed wide confidence intervals with a slight variance of results (Figure 2).

### 3.4 Publication bias

#### 3.4.1 Funnel plot

**Figure 3:**
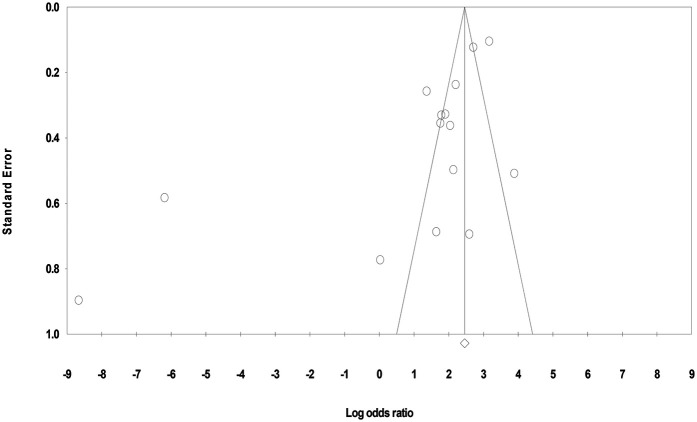
Funnel plot symmetry analysis for included studies.

The funnel plot appeared visually symmetrical; however, closer inspection revealed mild asymmetry, suggesting potential publication bias due to varying sample size between and within the studies and different types of sample analyzed. Under the fixed-effect model, the point estimate and 95% confidence interval for the combined studies was 11.67 and under the random-effects model the point estimate and 95% confidence interval for the combined studies was 2.69. Using Trim and Fill, these values remained unchanged (Figure 3).

#### 3.4.2 Eggers' regression intercept

Egger's test (*T* = 3.05, *p* = 0.009) further supported the publication bias. Thus, despite the visual appearance in the funnel plot, statistical results suggested small-study effects or selective publication, warranting cautious interpretation of the findings.

#### 3.4.3 Begg and Mazumdar’s rank correlation

Begg and Mazumdar's test yielded a Kendall's tau of −0.25 with a *p*-value of 0.18, indicating no significant rank correlation.

## 4 Discussion

Periodontitis is an inflammatory disease that causes periodontal tissue destruction, often linked to rheumatoid arthritis (5). Dickkopf-1 plays a central role in bone remodeling by inhibiting the Wnt signaling pathway, suppressing osteoblast function, and promoting osteoclast-mediated resorption. This disruption contributes to alveolar bone loss in periodontitis and joint erosion in rheumatoid arthritis (52, 53, 54). Pro-inflammatory cytokines, such as TNF-α and IL-1β, upregulate Dickkopf-1 expression, worsening tissue damage (55). Elevated Dickkopf-1 levels in serum and GCF serve as potential biomarkers for bone resorption and disease activity. These findings underscore Dickkopf-1 importance as both a diagnostic and therapeutic target (27).

This systematic review and meta-analysis assessed Dickkopf-1 as a biomarker for periodontitis, rheumatoid arthritis, and their comorbidity. Various studies have shown a significant impact of Dickkopf-1 in the progression of both periodontitis and rheumatoid arthritis. Diarra et al. showed that TNF-α stimulates Dickkopf-1 production linking inflammation to bone resorption (55). Vargas et al. found elevated serum Dickkopf-1 levels were a marker for joint damage in rheumatoid arthritis (56). De Pablo et al. suggested a bidirectional relationship between rheumatoid arthritis and periodontitis (57), while Kharlamova et al. highlighted *P gingivalis* in rheumatoid arthritis exacerbation (58). Ceccarelli et al. identified shared inflammatory mechanisms and genetic factors between both the diseases (59). Romero-Sánchez et al. linked elevated Dickkopf-1 in GCF to bone loss in periodontitis (27). Seror et al. found Dickkopf-1 predictive of rheumatoid arthritis structural progression, positioning it as a potential diagnostic and therapeutic target (44). Goes et al. identified Dickkopf-1 as a mediator of bone resorption in periodontitis through inhibition of osteoblast activity and promotion of osteoclastogenesis in mice model (60). Similarly, Buckland demonstrated the pivotal role of Dickkopf-1 in rheumatoid arthritis, particularly in joint destruction and inflammation (61).

Our analysis identified consistently elevated levels of Dickkopf-1 in patients with periodontitis and rheumatoid arthritis compared to healthy controls, as reported in 12 out of 15 studies, with an OR >1 and *p*-values <0.05, indicating a strong association of Dickkopf-1 with these conditions as a pro-inflammatory mediator. Another study carried out by Wu et al. observed Dickkopf-1 levels in periodontitis patients, which was non-significant, with an OR of 1.03 and a *p*-value >0.05, potentially attributable to the small sample size of 11 periodontitis patients in his study, limiting the strength of the correlation (40). On the other hand, two studies reported slightly reduced serum Dickkopf-1 levels in rheumatoid arthritis and periodontitis patients compared to healthy controls (39, 45). The dual role of Dickkopf-1 as an anti-oncogenic marker and a regulator of bone resorption may explain the decreased levels observed in periodontitis patients (39). Another study found reduced Dickkopf-1 levels in rheumatoid arthritis patients after anti-TNF α therapy (45).

Among the 15 studies reviewed, Jia et al. reported an odds ratio of 49 in periodontitis patients, indicating a strong significant association with Dickkopf-1 (41). Similarly, notable findings were observed in studies related to rheumatoid arthritis: Nocturne et al. demonstrated an odds ratio of 23, Seror et al. reported an odds ratio of 15, and Świerkot et al. documented an odds ratio of 13 (42, 44, 47). These findings highlight that Dickkopf-1 is highly associated with both periodontitis and rheumatoid arthritis. Since both conditions were characterized as bone-related diseases, the pivotal role of Dickkopf-1 in bone metabolism and pathology underscores its importance in the progression and regulation of these disorders. Our findings also indicate that Dickkopf-1 exhibits a significant association with periodontitis and rheumatoid arthritis, both as independent conditions and in their coexistence as comorbidities.

In the present meta-analysis on the role of Dickkopf-1 specifically in periodontitis alone, as well as its involvement in the context of comorbid periodontitis and rheumatoid arthritis, it was observed that elevated levels of Dickkopf-1 were consistently observed in studies by Jia et al., Jin et al., and Cardona-Rincón et al., suggesting its role as a pro-inflammatory marker that regulates bone remodeling by suppressing osteoblast activity and stimulating osteoclastogenesis (26, 38, 41). Its levels were elevated in periodontitis, linking inflammation to alveolar bone resorption, thereby positioning it as a potential diagnostic marker for periodontal disease. Dickkopf-1 acts by inhibiting the Wnt signaling pathway, enhances osteoclast activity, and accelerates bone degradation, a process observed in both periodontitis and rheumatoid arthritis. The increased expression of Dickkopf-1 contributes to the progression of alveolar bone loss and joint destruction, underscoring its role in comorbid conditions and highlighting its potential as a therapeutic target for mitigating bone loss.

In our study, the Q-value (473.9, *p* < 0.0001) and *I*^2^ (97%) indicate substantial heterogeneity among the included studies. Although Tau^2^ (0.753) reflects the between-study variance, Tau (0.868) gives the standard deviation of true effect sizes, suggesting moderate variability in effect sizes across studies. These values justify the use of a random-effects model and have been clearly explained to aid interpretation of the pooled effect size and enhance result transparency. Egger's test (*T* = 3.05, *p* = 0.009) revealed significant publication bias, suggesting both small-study effects and underreporting of studies with null results. This bias may inflate the pooled effect size and compromise the credibility and generalizability of Dickkopf-1 as a biomarker, highlighting the need for cautious interpretation, inclusion of gray literature, and further prospective research.

In the present study, the substantial heterogeneity observed in Dickkopf-1 levels across studies likely arises from methodological and population-related differences. Variations in disease definitions for periodontitis and rheumatoid arthritis, including the use of differing diagnostic criteria (e.g., CAL/PD vs. CDC/AAP for periodontitis; 1987 ACR vs. 2010 ACR/EULAR for rheumatoid arthritis), may have affected disease classification and Dickkopf-1 expression. Population diversity in terms of age, ethnicity, genetic background, and systemic health status further contributes to biomarker variability. Differences in sample types (serum vs. gingival crevicular fluid) introduce additional variability, reflecting systemic vs. localized inflammation. Geographic variation and environmental influences, along with differences in ELISA protocols (e.g., kit sensitivity, antibody specificity, and calibration methods), may also impact measured Dickkopf-1 levels. These factors collectively limit the comparability of findings and highlight the need for standardized diagnostic criteria and assay methodologies in future research.

Dickkopf-1 plays a pivotal role in bone metabolism by inhibiting the Wnt signaling pathway, thereby suppressing osteoblast activity and promoting osteoclast-mediated bone resorption. This mechanism underlies its involvement in both alveolar bone loss in periodontitis and joint destruction in rheumatoid arthritis. Pro-inflammatory cytokines, such as TNF-α and IL-1β, upregulate Dickkopf-1 expression, exacerbating tissue degradation. Several studies have identified elevated levels of Dickkopf-1 in serum and GCF as potential biomarkers of disease activity and bone resorption in both conditions. For example, Diarra et al. demonstrated that TNF-α induces Dickkopf-1 production, linking inflammatory signaling to bone loss (55). However, the meta-analysis revealed substantial heterogeneity (*I*^2^ = 97%, Q = 473.9, *p* < 0.0001), likely due to differences in study designs, sample sources, geographic variation, and diagnostic criteria, despite relatively low between-study variance (Tau^2^ = 0.753). Although these findings highlight the clinical relevance of Dickkopf-1 as a biomarker and possible therapeutic target, caution is warranted in interpretation due to methodological inconsistencies and potential publication bias.

This meta-analysis highlights several limitations that warrant consideration. The limited number of available studies examining the association between Dickkopf-1, periodontitis, and rheumatoid arthritis constrained the scope of analysis, particularly restricting the feasibility of subgroup analyses due to insufficient data on studies focused exclusively on periodontitis or its comorbidity with rheumatoid arthritis. Most included studies were cross-sectional or case–control in design, limiting causal inference, and none explored longitudinal outcomes. Significant heterogeneity (*I*^2^ = 97%, Q = 473.9, *p* < 0.0001) was observed across studies, likely attributable to differences in diagnostic criteria, population demographics, geographic origin, sample types (serum vs. gingival crevicular fluid), and variability in ELISA protocols, including assay sensitivity and manufacturer-specific differences. Furthermore, Dickkopf-1 assessment was limited to serum and GCF, with a lack of data on synovial fluid or saliva due to sparse literature, which may have offered broader insights into its biomarker potential. Although Egger's test indicated significant publication bias (*p* = 0.009), the exclusion of non-English studies and gray literature may further contribute to this bias. In addition, the geographic and ethnic diversity of study populations introduces variability that may impact generalizability. Collectively, these limitations underscore the need for well-designed, longitudinal, and mechanistic studies with standardized methodologies to clarify the clinical utility of Dickkopf-1 as a biomarker and therapeutic target in both periodontitis and rheumatoid arthritis.

In conclusion, the present meta-analysis highlights the potential involvement of Dickkopf-1 in the interplay between periodontitis and rheumatoid arthritis. Although elevated Dickkopf-1 levels in serum and GCF may indicate its relevance in these chronic inflammatory conditions, the findings should be interpreted with caution due to the observational design of the included studies and the presence of publication bias. At this stage, Dickkopf-1 can be considered a promising, but not yet established, biomarker or therapeutic target. Future research should focus on prospective cohort studies, investigation of Dickkopf-1 levels in additional biofluids such as synovial fluid and saliva, and clinical studies evaluating the impact of modulating Dickkopf-1 in therapeutic contexts to substantiate its clinical applicability.

## Data Availability

The original contributions presented in the study are included in the article/Supplementary Material, further inquiries can be directed to the corresponding authors.
