# Supplementary material for: Evaluating Dickkopf-1 as a biomarker: insights into periodontitis, rheumatoid arthritis, and their comorbidity—a systematic review and meta-analysis
Source: Front Dent Med. 2025 Jul 21;6:1593218. doi: 10.3389/fdmed.2025.1593218 (PMC12318980; doi:10.3389/fdmed.2025.1593218)
Supplement: Supplementary file 1 [file Datasheet1.pdf]

Supplementary File 1: Reproducible Search Strategy

**Section**	**Details**
**Search Period**	June 1, 2024 – December 31, 2024
**Authors Involved**	BM, JM, DP (search conducted independently)
**Databases Searched**	PubMed<br>Web of Science<br>Cochrane Library
**Gray literature sources**	Google Scholar<br>ResearchGate<br><br>Purpose: Screening of gray literature (e.g., preprints, non-indexed studies). These platforms lack structured indexing and peer-review; thus, findings were interpreted with caution.
**Additional Search**	Manual reference checks from bibliographies of key reviews and included articles

---

Search Terms and Logic

**Concepts Combined Using Boolean Operators (AND/OR)**	Keywords / MeSH Terms
**Rheumatoid Arthritis**	"Rheumatoid Arthritis" OR "Arthritis"[MeSH]
**Chronic Periodontitis / Periodontitis**	"Chronic Periodontitis" OR "Periodontitis" OR "Periodontal Disease" OR respective MeSH terms
**Dickkopf-1 (Dkk-1)**	"Dickkopf-1" OR "Dkk-1" OR "Dickkopf-1 Protein"[MeSH] OR "DKK-1"[MeSH]

Database-Specific Search Strings

Database	Search Strategy
**PubMed**	("Rheumatoid Arthritis"[MeSH] OR "Arthritis") AND ("Chronic Periodontitis"[MeSH] OR "Periodontitis"[MeSH] OR "Periodontal Diseases"[MeSH] OR "Chronic Periodontitis"[Title/Abstract] OR "Periodontitis"[Title/Abstract]) AND ("Dickkopf-1 Protein"[MeSH] OR "DKK-1"[MeSH] OR "Dickkopf-1"[Title/Abstract] OR "Dkk-1")
**Web of Science**	TS=("Rheumatoid Arthritis") AND TS=("Chronic Periodontitis" OR "Periodontitis" OR "Periodontal Disease") AND TS=("Dickkopf-1" OR "Dkk-1")
**Cochrane Library**	("Rheumatoid Arthritis" in Title Abstract Keyword) AND ("Chronic Periodontitis" OR "Periodontitis" OR "Periodontal Disease" in Title Abstract Keyword) AND ("Dickkopf-1" OR "Dkk-1" in Title Abstract Keyword)
**Google Scholar**	"Rheumatoid Arthritis" AND ("Chronic Periodontitis" OR "Periodontitis" OR "Periodontal Disease") AND ("Dickkopf-1" OR "Dkk-1")
**ResearchGate**	Manual search using: “Dickkopf-1” AND “Periodontitis” AND “Rheumatoid Arthritis”

Eligibility Criteria

Inclusion Criteria	Exclusion Criteria
-Human studies<br>- Focus on Dkk-1 in context of RA, periodontitis, or both	- Duplicates<br>- Animal studies<br>- In vitro studies

Inclusion Criteria	Exclusion Criteria
- ELISA-based molecular analysis<br>- Full-text in English	- Unrelated conditions without Dkk-1 as primary biomarker

PRISMA Flow Summary (Refer to Figure 1)

Stage	Number of Records
**Total Records Identified**	386
**Duplicates Removed**	163
**Records Screened**	223
**Full-text Articles Assessed for Eligibility**	89
**Studies Included for Qualitative Synthesis**	15 (selected based on Newcastle-Ottawa Scale)
