# Supplementary material for: Evaluating Dickkopf-1 as a biomarker: insights into periodontitis, rheumatoid arthritis, and their comorbidity—a systematic review and meta-analysis
Source: Front Dent Med. 2025 Jul 21;6:1593218. doi: 10.3389/fdmed.2025.1593218 (PMC12318980; doi:10.3389/fdmed.2025.1593218)
Supplement: Supplementary file 2 [file Datasheet2.pdf]

Newcastle-Ottawa Scale Domain-wise Scores for Included Studies

Sl.no	Author and Year	NOS Score	Selection (0–4)	Comparability (0–2)	Outcome/Exposure (0–3)
1	Cardona-Rincon (2020)	4	2	1	1
2	Antohi C (2022)	7	4	2	1
3	Jing J (2016)	4	2	1	1
4	Wu KY (2017)	6	3	1	2
5	Jia H (2022)	6	3	1	2
6	Nocturne G (2015)	6	3	1	2
7	Wang SY (2010)	7	4	2	1
8	Seror R (2015)	6	3	1	2
9	Daoussis D (2010)	6	3	1	2
10	Fassio A (2017)	6	3	1	2
11	Swierkot J (2015)	6	3	1	2
12	Rossini M (2015)	6	3	1	2
13	Choi BY (2014)	6	3	1	2
14	Long L (2010)	6	3	1	2
15	Voorzanger (2009)	4	2	1	1
